# The feasibility, facilitators, and barriers in the initial implementation phase of ‘good life with osteoarthritis in Denmark’ (GLA:D®) in Switzerland: a cross-sectional survey

**DOI:** 10.1186/s12913-023-10023-7

**Published:** 2023-09-27

**Authors:** Anja Hinteregger, Karin Niedermann, Markus Wirz

**Affiliations:** School of Health Sciences, Institute of Physiotherapy, ZHAW Zurich University of Applied Sciences, Katharina-Sulzer-Platz 9, Winterthur, 8400 Switzerland

**Keywords:** Osteoarthritis, Health education, Exercise therapy, Non-surgical management, Implementation, Feasibility, Survey

## Abstract

**Background:**

The guideline-based, conservative, non-pharmacological management of hip and knee osteoarthritis in clinical practice has been insufficient in Switzerland until now. The implementation of “Good Life with Osteoarthritis in Denmark” (GLA:D®), a programme designed to address this evidence-performance gap, was started in 2019 in Switzerland. This study investigated the acceptance and practicality of the GLA:D® Switzerland programme and identified the facilitators and barriers to its implementation, to support the development of tailored implementation strategies.

**Methods:**

This is a non-experimental observational study. A cross-sectional survey was performed among the physiotherapists (PTs) of the first five GLA:D® Switzerland certification courses, using the Measurement Instrument for Determinants of Innovations (MIDI) to identify the facilitators and barriers. Descriptive statistics were calculated, and qualitative content analysis was used for open-ended questions.

**Results:**

In the online survey, 86 GLA:D® certified PTs participated (response rate: 61%). The majority of 51 PTs (63.7%) worked in private practices. Of the responding PTs 58 (78.4%) were satisfied with the general concept of the GLA:D® Switzerland programme. Practicality was evaluated positively, particularly the second and third individual session (n = 40 PTs, 83.3%), the 40 m Fast-paced Walk Test (43, 89.6%), the 30 s Chair Stand Test (45, 93.8%), and the exercise programme (40, 83.3%). The marketing (12, 15%), the ‘data entry’ (5, 10.4%), ‘register the patient’ (7, 14.6%), and the digital patient questionnaire (9, 14.2%) were rated less positively. In total, 12 facilitators and 12 barriers were identified. The barriers were mainly related to adopting user, e.g., perceived personal disadvantages. Barriers were also found in the organisational context, e.g., time available. Facilitators were associated with the GLA:D® Switzerland programme itself, e.g., completeness, relevance for patients, and the adopting user, e.g., self-efficacy, and in the organisational context, e.g., material resources and facilities. Topics related to the socio-political context were raised in the answers to the open-ended questions, e.g., general awareness level of the GLA:D® Switzerland programme and patient recruitment.

**Conclusion:**

The acceptance, practicality and facilitators identified from the initial implementation are encouraging. However, the identified barriers and activities rated with low practicality require tailored strategies to support a successful implementation of the GLA:D® Switzerland programme.

**Supplementary Information:**

The online version contains supplementary material available at 10.1186/s12913-023-10023-7.

## Background

Osteoarthritis (OA) is the most common joint disease worldwide and a major cause of chronic musculoskeletal pain and disability [1]. As well as having various personal, social and financial consequences for patients, it also accounts for a significant economic burden on society [2–4].

Based on consistent and high-quality evidence for its effectiveness and safety, international clinical guidelines recommend patient education, exercise, and weight management, if appropriate, as first-line treatment for hip and knee OA [5–8]. However, investigations in many countries indicate that the transfer of these guideline recommendations into clinical practice, and therefore, the conservative, non-pharmacological management of hip and knee OA, is insufficient [9–13]. Also in Switzerland, this evidence-performance gap is present [14].

‘Good Life with Osteoarthritis in Denmark’ (GLA:D®) was designed to address this gap [15]. It is a standardised treatment programme for hip and knee OA patients based on the clinical guideline recommendations. The GLA:D® programme is offered by certified physiotherapists (PTs) after a two-day certification course. The GLA:D® Switzerland programme consists of four individual sessions, two one-hour patient education sessions and 12 supervised, personalised neuromuscular exercise one-hour group sessions with usually three to five participants twice a week. The third essential feature is the collection of patient characteristics and clinical assessments in a national data register at start and end of treatment and at twelve-month follow-up. After concluding the post-treatment assessments, a report to the referring physician is generated. This documentation allows reporting of the individual changes as well as quality control of the GLA:D® programme [16].

According to the GLA:D® Denmark Annual Report 2021, around 10,000 patients annually have participated in the programme in Denmark alone since 2013 [17]. These participants showed considerably lower pain intensity, less use of painkillers, better function and reduced sick leave days, as well as improved quality of life at the three-month and twelve-month follow-ups [16].

Since its initiation in 2013 in Denmark, the GLA:D® programme has been implemented in other countries, such as Canada, Australia and China [15] and in 2019 in Switzerland [18]. The country-specific context, such as the involvement of the right healthcare providers or organisation of the healthcare system, must be taken into account for a successful implementation [19, 20]. The GLA:D® programme, particularly the guideline-based, standardised management of hip and knee OA, and the documentation of clinical outcomes in a national data register, is novel for PTs in Switzerland. The first GLA:D® certification courses for PTs in Switzerland took place in April and May 2019. Those participants can be considered early adopters, who are generally more open to innovation and changes and therefore an especially motivated group [21].

Research into the implementation of an innovation highlights the importance of monitoring and guiding the different phases of the process [19, 22–29]. At the time of this study, the GLA:D® Switzerland programme was in its initial implementation phase [23] which is dynamic and requires changes at various context levels (e.g. individual or practice environment) [23]. This was an appropriate opportunity to evaluate the feasibility and perceived facilitators and barriers among the early adopters in order to learn and support the implementation process and the PTs adopting the programme.

There are several determinants, i.e. (facilitators and barriers) that can affect these changes and the success of an implementation. The identification of facilitators and barriers among the relevant stakeholders is an important step in designing successful implementation strategies [19, 30]. Facilitators and barriers are related to: (1) the characteristics of the innovation (e.g. complexity, compatibility); (2) the user (e.g. personal drawbacks, social support, knowledge); (3) the patient (e.g. cooperation, satisfaction, health status); (4) the organisational context (e.g. material or financial resources, staff capacity, unsettled organisation); or the (5) socio-political context (legislation and regulations) [19, 23, 29, 31].

Consequently, the aim of this study was to investigate the feasibility, such as acceptance and practicality, of the GLA:D® Switzerland programme and to identify the facilitators and barriers experienced by certified PTs, in this initial implementation phase. The results will guide and support the ongoing implementation of the GLA:D® programme in Switzerland.

## Methods

### Study design

This is a non-experimental, cross-sectional observational study.

### Participants and recruitment

The PTs from the first five GLA:D® Switzerland certification courses in the French-, Italian- and German-speaking regions of Switzerland were surveyed between November 2019 and March 2020. PTs who had attended the certification course but had not yet implemented a programme for patients themselves were also included in the survey.

### Content and development of the survey

An online survey, including both open-ended and closed-ended questions, was used for data collection(Additional file 1). It was composed of questions on demographics and professional characteristics, as well as on the feasibility, and items of the “Measurement Instrument for Determinants of Innovations” (MIDI) [30] for the identification of barriers and facilitators. A total of 84 items were included.

The online survey was created with the Unipark programme of the Questback GmbH, using the Enterprise Feedback Suite Survey software [32]. All items were displayed in an unaltered, standardised order, and filter functions were used to improve efficiency. For example, participants who had not yet conducted a programme for patients could skip inapplicable questions.

A pilot test of the online survey was performed with five GLA:D® Switzerland certified PTs. Upon completion of the pilot-testing, adaptations were made to abbreviate the online survey and to ensure face validity, clarity, plausibility, and completeness.

#### Feasibility

The questions related to feasibility focused on acceptance (two items) and practicality (18 items), as described by Bowen et al. [33]. The section concerning acceptance included two closed questions on satisfaction and the intention to continue using the programme. The section concerning practicality was composed of 18 closed questions on the various components of the GLA:D® Switzerland programme. A five-point Likert scale was used to rate the practicality items, ranging from ‘very poor’ to ‘very good’. For activities that had not yet been carried out, the option ‘I have not done it yet’, was added to the answer scale.

In addition, a total of seven open-ended questions were included, asking for comments, suggestions for improvement, and explanatory statements.

#### Facilitators and barriers

The MIDI [30] was used to identify potential facilitators and barriers to the implementation of the GLA:D® Switzerland programme. This outcome measure was developed by Fleuren et al. [30] to improve the understanding of factors that influence the implementation of an innovation.

The MIDI comprises 29 items that can be categorised into four domains: (1) innovation; (2) adopting user (including two factors related to patient characteristics); (3) organisational context; and (4) socio-political context. In this study, the innovation refers to the GLA:D® Switzerland programme, the adopting users are the certified PTs, the organisational context is the individual working environment, and the socio-political context refers to the socio-political context of Switzerland. In particular, the organisational context includes outpatient practices, clinics, hospitals, rehabilitation clinics and other, e.g., interprofessional health centres, as well as institutions for chronically ill.

A five-point Likert response scale is used for most items of the MIDI, ranging from, e.g., ‘totally disagree’ to ‘totally agree’.

#### Open-ended questions

Three general open-ended questions, based on questions used in the interview guide of the implementation feasibility study of GLA:D® Canada, were included [34]. The questions asked were: (1) what worked well; (2) what challenges were encountered; and (3) what recommendations the PTs would make to others.

### Data collection

The PTs received an invitation to participate in the survey between four and seven months after completing the GLA:D® Switzerland certification courses. The invitation was sent by e-mail and included a link to the online survey. This four-month time period after the certification courses was chosen to allow PTs to begin offering the GLA:D® Switzerland programme to their patients. Reminder e-mails were sent after two and three weeks.

### Data processing and analysis

Descriptive statistics were calculated as means ± standard deviations (SD), medians + interquartile ranges (IQR) and frequency (proportions), as appropriate. All answers, including those from incomplete surveys, were eligible for data analysis. Data analysis was made using Microsoft Excel Office 365, 2016 and IBM SPSS version 26.0 [35].

#### Facilitators and barriers

The answer options ‘agree’ and ‘totally agree’, and ‘totally disagree’ and ‘disagree’ respectively, were collapsed to perform the analysis. After consultation with Dr. M. Fleuren, MIDI items with answers of ≥ 20%, ‘totally disagree / disagree’, were considered to be barriers and those with responses of ≥ 80%, ‘agree / totally agree’, to be facilitators.

#### Open-ended questions

The open-ended questions were analysed by means of a qualitative content analysis. A coding frame was created by combining a concept-driven (deductive) and data-driven (inductive) approach [36]. Firstly, the four main categories of ‘acceptance’, ‘practicality’, ‘facilitators’ and ‘barriers’, and their related subcategories, were defined deductively. The coding frame was then tested, discussed, and revised by two researchers and the main category ‘Other’, as well as further subcategories, were added inductively. Finally, the absolute frequency of subcategories was presented, and the most frequent subcategories were described using continuous text.

## Results

A total of 141 PTs attended the first GLA:D® Switzerland certification courses and were invited to participate in the online survey. Of these, 86 participated in the survey, resulting in a response rate of 61%. Of the 86 participants, 65 (75.6%) had fully completed the questionnaire and 27 (31.4%) had not yet conducted a programme for patients. Further demographic and professional characteristics of the respondents are presented in Table 1, with comprehensive details provided in Additional file 2.

The absolute frequencies of the subcategories of the coding matrix for the open-ended questions are given in Additional file 3.

**Table 1:** Characteristics of responding PTs

Characteristic	No. (%)	Mean ± SD (range)
**Sex (n = 65)**		
female	40 (61.5%)	
male	25 (38.5%)	
**Age in years (n = 63)** *		42.6 ± 10.8 (26–67)
**Language (n = 85)**		
German	58 (68.2%)	
French	11 (12.9%)	
Italian	16 (18.8%)	
**Work experience in years (n = 65)**		16.5 ± 10.7 (1–44)
**Highest degree of education (n = 65)**		
No bachelor’s degree or subsequent title acquisition ^b^	11 (16.9%)	
Subsequent title acquisition ^b^	15 (23.1%)	
Bachelor of Science	22 (33.8%)	
Master of Science	12 (18.5%)	
Doctorate	1 (1.5%)	
Other	4 (6.2%)	
**Place of work (n = 65)** ^**a**^		
Outpatient practice	51 (63.7%)	
Clinic / Hospital	17 (21.3%)	
Rehabilitation clinic	3 (3.8%)	
Interprofessional health centre	4 (5.0%)	
Institution for chronically ill	1 (1.3%)	
Other	4 (5.0%)	
**Number of PTs working in the institution (n = 63)**		12.1 ± 17.6 (1–80)
**Professional position (n = 65)** ^**a**^		
Employed physiotherapist	35 (47.3%)	
Practice trainer / Medical practice trainer	3 (4.1%)	
Specialist manager / Professional content expert	2 (2.7%)	
Management function	5 (6.8%)	
Practice owner	25 (33.8%)	
Management board / Clinic management	1 (1.4%)	
Other	3 (4.1%)	
**GLA:D® Switzerland Function (n = 81)**		
GLA:D® Switzerland trainer ^c^	7 (8.6%)	
GLA:D® Switzerland PTs	74 (91.4%)	
**Participated certification course (n = 65)**		
April 2019 (German-speaking Switzerland)	15 (23.1%)	
May 2019 (German-speaking Switzerland)	11 (16.9%)	
September 2019 (German-speaking Switzerland)	25 (38.5%)	
September 2019 (Italian-speaking Switzerland)	9 (13.8%)	
October 2019 (French-speaking Switzerland)	5 (7.7%)	
**Started a programme for patients (n = 80)**		
Yes	53 (66.3%)	
No	27 (33.8%)	
**Number of**:		
started programmes for patients (n = 44) *, **		2.1 ± 1.8 (1–10)
patients participated (n = 49)		9.1 ± 8.9 (1–56)
patients with medically prescribed therapy (n = 40) *		9.3 ± 9.1 (0–54)
self-pay patients (n = 26)		2.0 ± 2.6 (0–11)

* One response was declared as missing because the answer was not explicit.

** One PT gave ‘started programmes for patients:30’ and ‘patients participated: 30’ as

a response. The answer ‘started programmes: 30’ was therefore declared as non-plausible.

^a^ Multiple answer possible

^b^ Since 2009, qualified physiotherapists in Switzerland have been able to acquire a subsequent university

of applied sciences title.

^c^ GLA:D® Switzerland trainers are PTs who are certified to give GLA:D® Switzerland certification courses to train

other PTs.

SD: standard deviation

### Feasibility

#### Acceptance

**Fig. 1:**
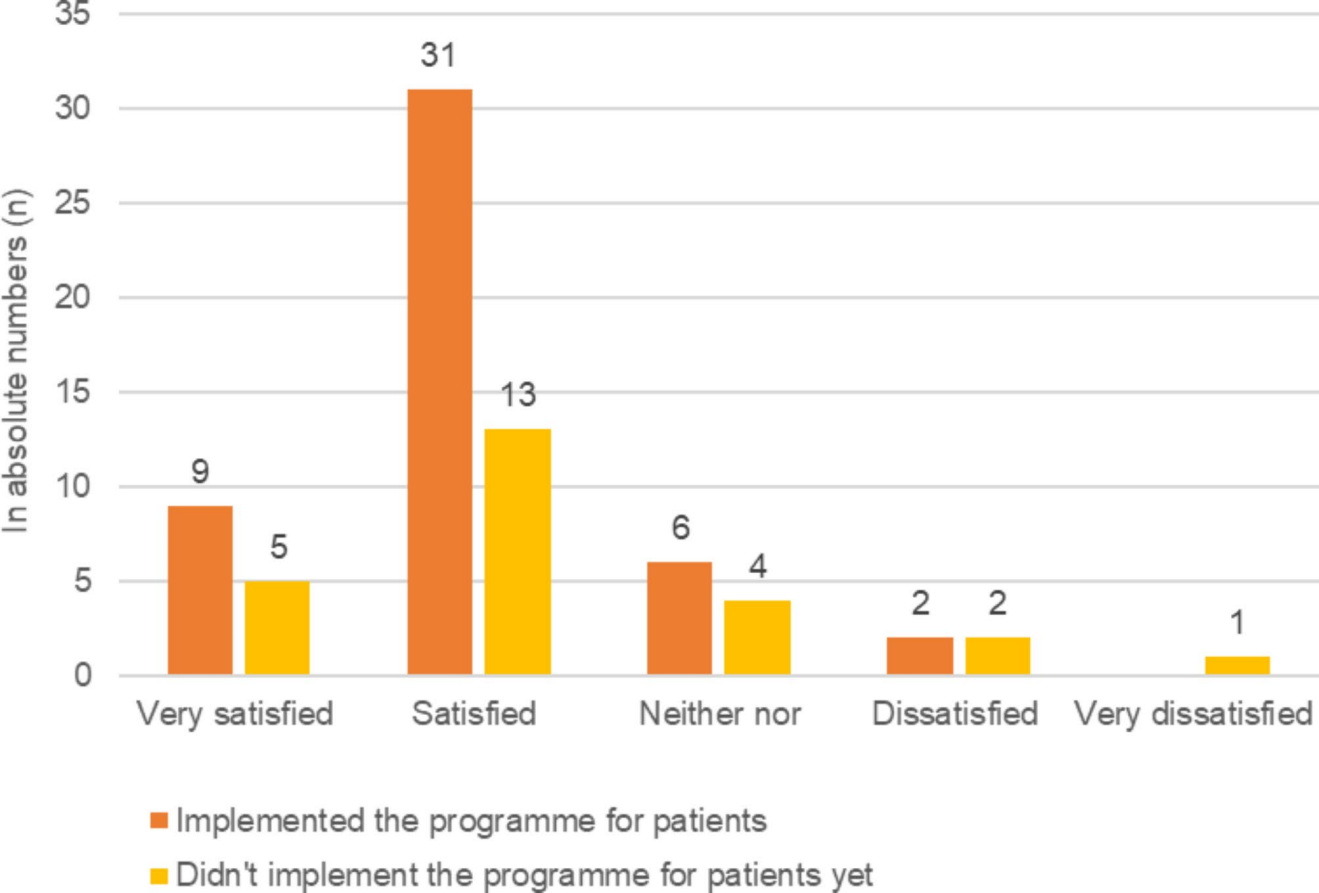
Satisfaction with general concept of GLA:D® Switzerland (n = 74), given in absolute numbers

Of the responding PTs, 58 (78.4%) were satisfied with the general concept of the GLA:D® Switzerland programme, whilst one third of the responses, 25 (33.8%), were from PTs who had not yet implemented the GLA:D® Switzerland programme for patients (Fig. 1).

The answers to the open-ended questions revealed that the PTs considered the GLA:D® Switzerland programme a good, reasonable, useful therapeutic approach, and that they appreciated the way the programme is designed (n = 8). Dissatisfaction was mainly expressed about the general effort required, the marketing effort, difficulties of patient recruitment, and the data register (n = 8).

Of the responding PTs, 68 (90.7%) intend to offer GLA:D® Switzerland programmes for their patients in the next six months, while seven (9.3%) PTs indicated that they do not intend to do so. Only a few PTs (n = 5) gave reasons for not intending to offer the GLA:D® Switzerland programme (Additional file 2).

#### Practicality

**Fig. 2:**
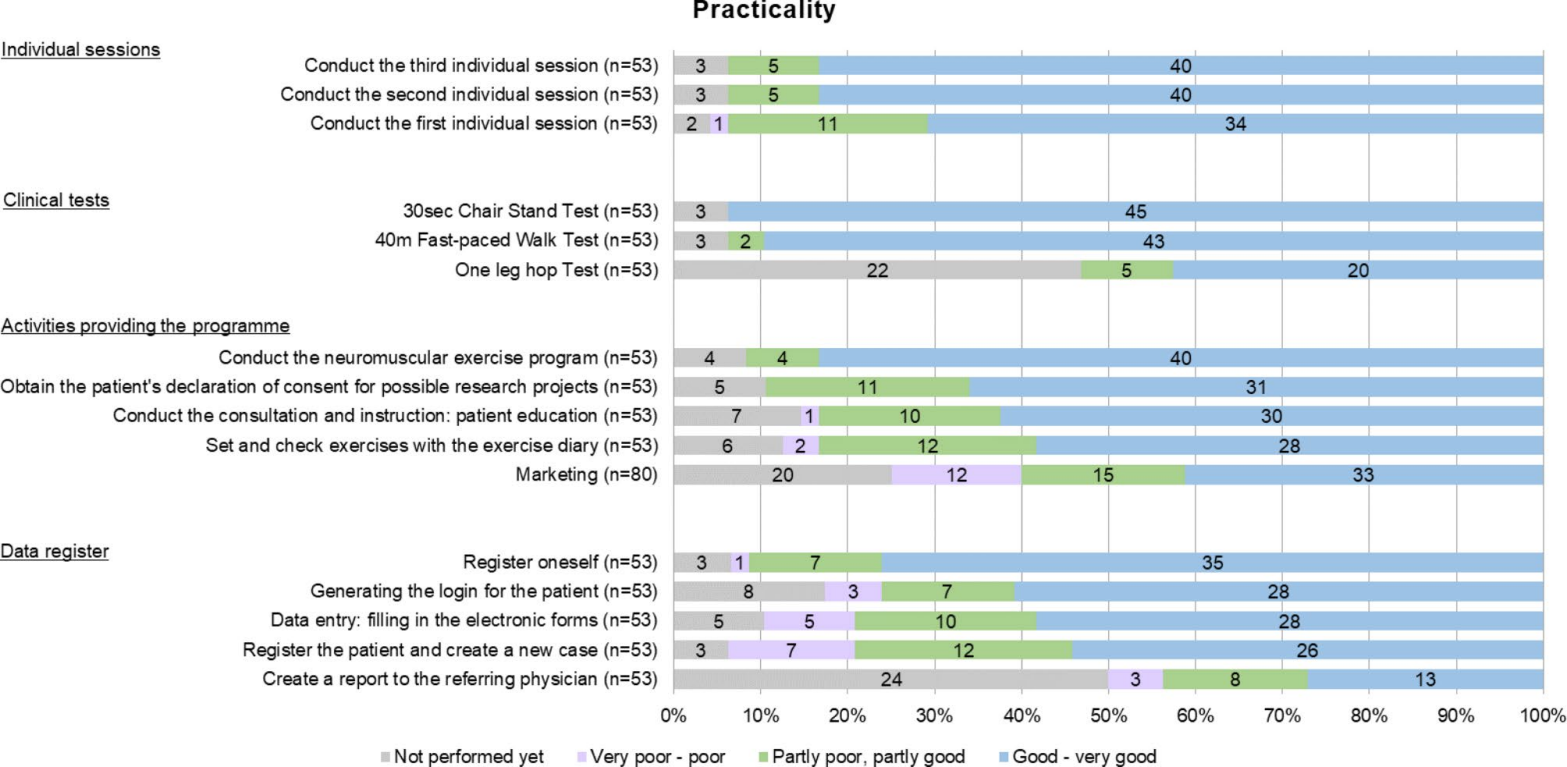
Practicality

The responses to the question, ‘How did you, as a physiotherapist, manage the following activities?’ are illustrated in Fig. 2. Filter functions were used in the survey, so the responses are only from PTs who have already implemented the programme 53 (66.3%) except for the item marketing.

Regarding the practicality of the digital patient questionnaire, nine (14.2%) rated its practicality as poor to very poor, 18 (28.6%) gave a neutral response, three (4.5%) rated it as good. Furthermore, 27 PTs (42.9%) stated that they had completed the digital patient questionnaire together with the patient. As well, the open-ended questions indicated that the PTs encountered difficulties with the digital patient questionnaire (n = 16) due to limited resources (facilities, time and material resources) and patient characteristics (age, cooperation, heterogeneity of groups, and digital skills).

The open-ended questions on practicality revealed that the general process and implementation of the GLA:D® Switzerland programme were feasible (n = 11). Many also reported positive experiences conducting the neuromuscular exercise programme (n = 15). However, some PTs mentioned difficulties with the time available to complete some tasks of the programme (e.g. neuromuscular exercise programme (n = 13), patient education (n = 6), individual sessions (n = 6)).

In addition, the PTs described the planning and organisation of the groups as challenging (n = 24): forming of patient groups, scheduling the exercise groups, patient education and individual sessions, efficient planning of groups, as well as the reorganisation of working hours and premises. In the open-ended questions, many PTs stated both, patient recruitment (n = 9) and marketing (n = 8) to be challenging.

Concerning the data register, the PTs named, technical difficulties and insufficient usability and practicality of the electronic data collection interface in the open-ended questions (n = 23).

### Facilitators and barriers

**Fig. 3:**
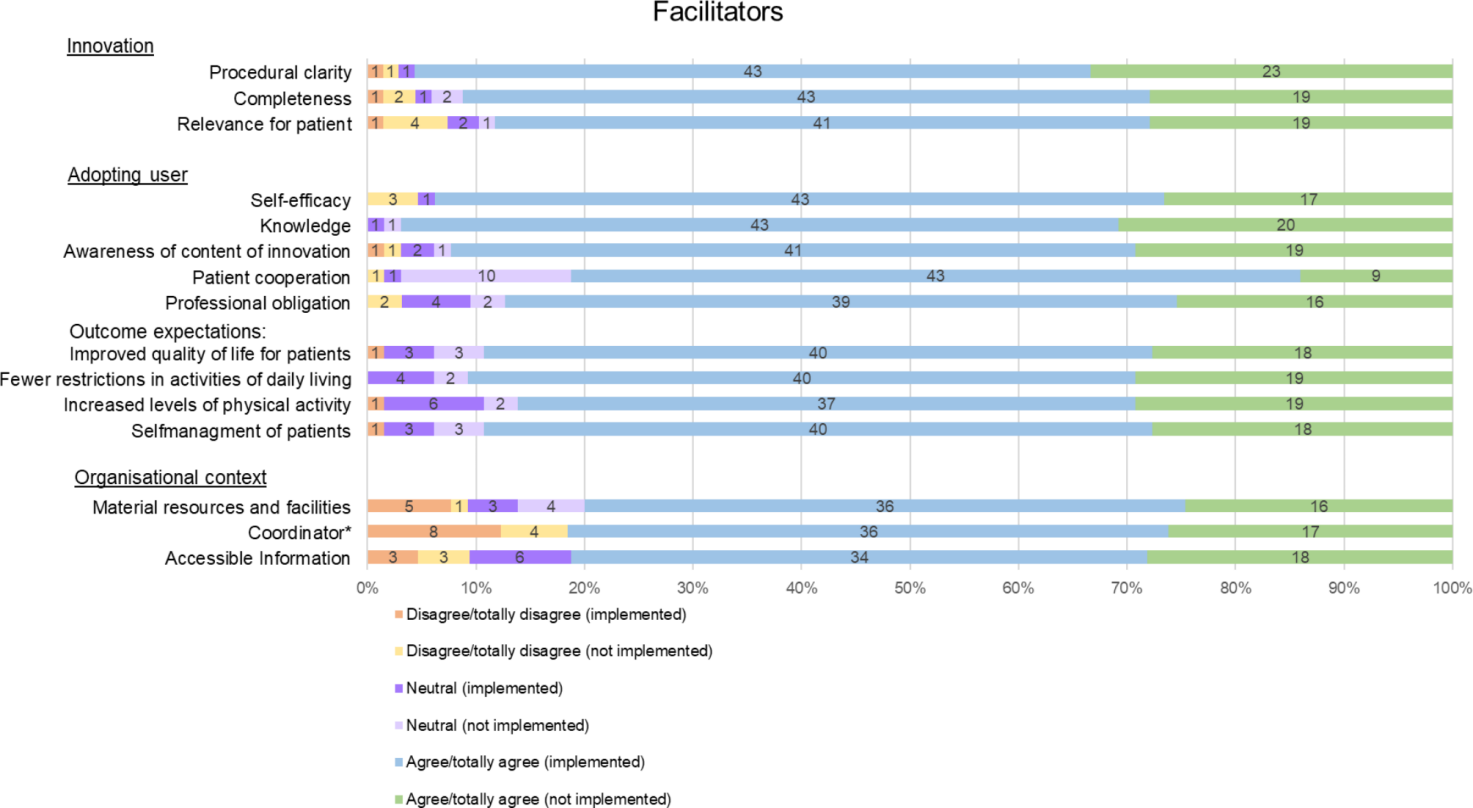
Facilitators. Outcome expectations are considered as one facilitator as described in the MIDI. *dichotomous answer categories

**Fig. 4:**
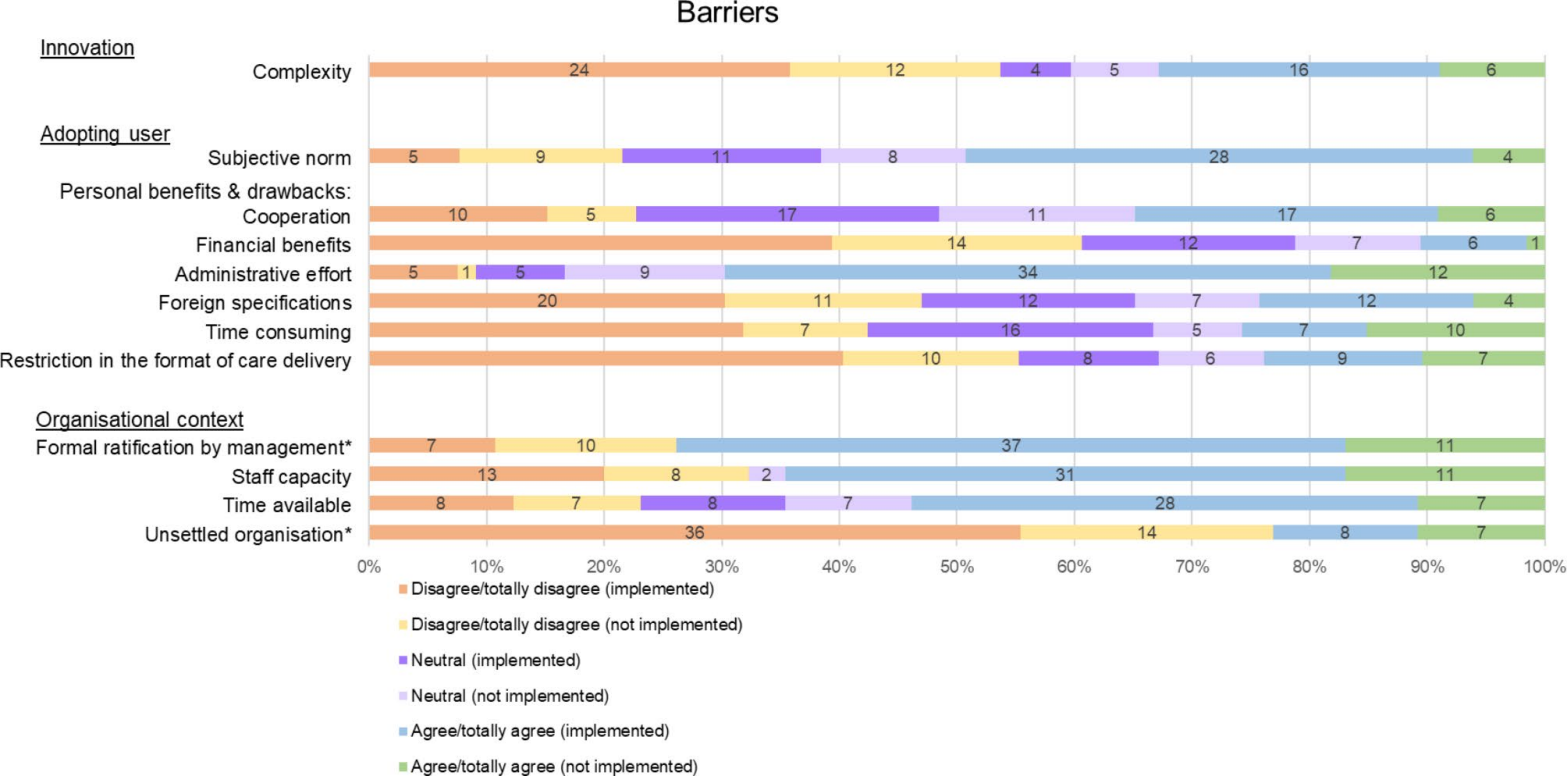
Barriers. *dichotomous answer categories

In total, 12 facilitators and 12 barriers to implementing the GLA:D® Switzerland programme were identified using the MIDI and its categories (Figs. 3 and 4). The detailed results of all items and their operationalization can be found in Additional file 4.

#### The innovation (GLA:D® Switzerland programme)

Regarding the innovation (the GLA:D® Switzerland programme), three facilitators were identified, namely (1) ‘procedural clarity’ (2) ‘completeness’ and (3) ‘relevance for patient’. ‘Complexity’ was identified as a barrier. Of the respondents, 22 (32.8%) agreed that there were components of the GLA:D® Switzerland programme that were too complex. The open-ended questions confirmed the most issues with the data register (n = 22): complexity or effort, usability, electronic data collection, evaluation, and the creation of a report.

#### The adopting user (GLA:D® Switzerland certified PTs, patients)

In association with the GLA:D® Switzerland certified PTs, a total of five facilitators and seven barriers were identified. ‘Cooperation’ is concerned with the facilitation of collaboration with other professionals through the GLA:D® Switzerland programme. Another barrier associated with the PTs was: the ‘subjective norm’, or perceived expectation of others. Some 32 (21.5%) of the PTs reported that their work environment did not expect them to implement the GLA:D® Switzerland programme.

One facilitator associated with the patient characteristics was ‘patient cooperation’. In the responses to the open-ended questions, several PTs mentioned patient motivation (n = 11) as facilitating (e.g., positive group dynamics). However, the open-ended questions also revealed barriers related to patient characteristics: age (n = 6); (digital) skills (n = 6); and patient cooperation (n = 11) (e.g., in planning the groups, active therapy, group setting, comprehension of patients).

#### The organisational context

Three items could be assigned to organisational facilitators and four to organisational barriers. In the open-ended questions, PTs commented several times that there were issues with limited resources (general, facilities, time, personnel, and material, n = 23). Moreover, lack of resources (n = 9) and unsettled organisation (n = 2) were given as reasons for the non-implementation of the GLA:D® Switzerland programme.

#### The socio-political context

The only item of the MIDI concerning the socio-political context ‘legislation and regulations’ could be classified neither as a facilitator nor as a barrier.

However, topics related to the socio-political context were raised several times in the responses to the open-ended questions. The PTs raised the issue of insufficient awareness of the programme, both generally and amongst medical doctors (n = 11). Moreover, they described low patient referrals (n = 4), an insufficient number of patients (n = 9), and difficulties in patient recruitment (n = 6). Insufficient numbers of patients were also given as a reason for the non-implementation of the GLA:D® Switzerland programme (n = 12). Profitability was a further topic mentioned several times (n = 16). For example, some of the PTs evaluated the group sessions to be financially profitable only with more than four or five patients (n = 5). In addition, a few PTs mentioned insufficient monetary compensation from the health insurance for the workload to be an issue (n = 5).

## Discussion

This study investigated the feasibility and facilitators of and barriers to the initial implementation phase of the GLA:D® programme experienced by certified PTs in Switzerland.

The PTs perceived the feasibility, acceptance and practicality, of the GLA:D® Switzerland programme as high. This, together with the identified facilitators, supports the further successful roll-out of the programme. The barriers were mainly related to the individual level, i.e., the certified PTs, and the organisational context. These, and the identified difficulties concerning practicality, require specific strategies.

### Feasibility

#### Acceptance

The participants gave noteworthy positive responses regarding acceptance, i.e., satisfaction and intention to continue using the GLAD programme. Nevertheless, it should be mentioned that, according to the Rogers’ adoption model, the participants in this study can be regarded as ‘early adopters’. These ‘early adopters’ are considered to be more motivated and are more likely to form favourable opinions about an innovation [21].

#### Practicality

Concerning practicality, the conduction of the clinical tests and neuromuscular exercise programme were rated as feasible by the PTs. Although the importance of using standardised assessments is generally acknowledged within the physiotherapy profession, their application in clinical practice is still insufficiently established [37, 38]. Several challenges to the use of standardised assessments within the physiotherapy profession have been described [37–40]. It is, therefore, even more remarkable that the application of standardised assessments in the GLA:D® Switzerland programme functioned well. The gathering and evaluation of assessments is an integral part of the GLA:D® Switzerland programme. There is a specific appointment for the initial and exit examination. Moreover, the GLA:D® Switzerland programme specifies which assessments are conducted and when they are performed. In addition, GLA:D® Switzerland are taught how to perform the assessments in the certification courses. This seems to be favourable for the implementation of standardised assessments.

The PTs experienced difficulties with data entry and patient data registration. A digital technology change, such as the introduction of the data register, requires awareness of the key aspects [41]. For the implementation of digital applications, examples of these key aspects are the knowledge and beliefs of the user, the compatibility of the digital application with the workflow, and its user-friendliness [42]. The provision of training, technical support and guidance to users and involving them in the development process of the new digital application is recommended [41–43].

Many PTs reported that they had to assist their patients with the completion of the digital patient questionnaire. The intention of GLA:D® Switzerland is that patients fill out the digital patient questionnaire independently. The PTs reported difficulties regarding the digital patient questionnaire due to the lack of available resources (facilities, time, and material resources) and patient characteristics (age, cooperation, heterogeneity of groups, and digital skills). Therefore, it would be essential to pay special attention to the necessary resources in the certification courses. In addition, strategies for managing patients with characteristics that are limiting for the digital questionnaire should be developed.

One quarter of PTs stated, they had not carried out any marketing, and some rated the practicality of the marketing as low. It is intended by GLA:D® Switzerland that the PTs undertake the marketing for the patient courses themselves. While the subject of marketing is covered in the GLA:D® Switzerland certification course, this finding suggests that more extensive support is needed and that the topic of marketing should be covered in more depth in the certification course.

### Facilitators and barriers

#### The innovation (GLA:D® Switzerland programme) and the adopting user (GLA:D® Switzerland certified PTs, patients)

In the implementation of recommendations for the treatment of OA in primary care, a lack of clarity, knowledge, and skills have been identified as barriers [27, 44]. However, in this study, the PTs’ knowledge, and the procedural clarity of the GLA:D® Switzerland programme were identified as facilitators. The PTs also held encouraging views of their self-efficacy, the relevance of the programme to their patients, and the patients’ outcome expectations. Self-efficacy and skills, in conjunction with perceived demand and the benefits of an innovation, were found to lead to greater implementation and fidelity [19, 24].

Additional effort and workload caused by the application of an innovation is regarded as acting as a barrier to implementation [28]. Personal drawbacks were identified for ‘administrative effort’ and ‘time expenditure’ in this study. PTs mentioned difficulties with the: time available for, and the extent of, the neuromuscular exercise programme; patient education; and the individual sessions. In addition, several PTs considered the administrative tasks and the planning and organisation of the individual and group sessions to be challenging. This is consistent with the literature on implementation, which indicates significant administrative changes during the initial implementation phase [23]. The additional effort described by the PTs could be attributed to the programme being in its initial implementation phase, which requires changes on several levels [23]. However, research has shown that individual perceptions change throughout the implementation process, with new work processes are initially viewed as a barriers but later becoming facilitators as workflow improves and the innovation is adopted [28]. Nevertheless, the importance of administrative support has been emphasised several times in the literature [24]. Therefore, there should be a greater focus on addressing administrative reorganisation and administrative support in the GLA:D® Switzerland certification courses.

In a feasibility study on the GLA:D™ Canada programme implementation, the PTs described how they had managed the scheduling of the classes successfully [34]. Initially, classes were scheduled at different times and days for flexibility and coverage by another PT. Once a week, the education sessions were offered, alternating between session one and two. New patient assessments and final testing were scheduled 30 min before supervised exercise classes. As demand increased, classes were offered six times per week, early morning and late afternoon, to control class size. For a successful execution, class size and the management of new participants were emphasised to be important. PTs of the GLA:D™ Canada programme indicated that initial classes should be small (three or four patients) and rolling recruitment was very beneficial, to gradual increase class size [34]. PT experiences from this study and the feasibility study on the GLA:D™ Canada programme could be incorporated into the GLA:D® Switzerland certification courses. In addition, strategies from PTs who have successfully mastered the initial phase of the GLA:D® Switzerland programme could be gathered and provided in the GLA:D® Switzerland certification courses.

Overall, patient cooperation was mostly seen as beneficial in the GLA:D® Switzerland programme, fostering positive dynamics and motivation. However, the open-ended questions revealed that in individual cases there were difficulties in patient cooperation.

#### The organisational context

Because the responding PTs worked in various work settings, the organisational context for the PTs varied to some degree. However, most PTs worked in an outpatient practice, clinic, or hospital. The findings concerning organisational facilitators and barriers are in line with those reported for OA care among clinicians [44] and for complex health interventions in primary care [28]. As an example, Grol et al. [19] provide a wide range of strategies to overcome such organisational barriers on the individual or team level, the organisational level, and at the level of the health system.

According to the findings of Lau et al. [28], financial issues can act as major facilitators or barriers. In the open-ended questions, the PTs concerns were related to lack of profitability, insufficient patient numbers, and difficulties in organising group sessions with enough participants. The perceived lack of profitability could be due to the initial implementation phase, which necessitates increased administrative effort and changes in the workflow. However, it might also be strongly related to the level of programme awareness, difficulties in conducting marketing, and insufficient patient referrals.

#### The socio-political context

The described difficulties relating to the socio-political context of insufficient patient enquiries and low number of referrals, might be associated with the current lack of awareness of the programme. However, another explanation may be that referral to physiotherapy is not routinely prescribed in the current practice of primary care management of OA. Referral to physiotherapy due to OA as a proportion of all OA cases managed by general practitioners is low [14, 45]. This is particularly the case when OA is a newly-diagnosed health problem [45]. In a survey of medical doctors in Switzerland, the participants estimated that they had referred only 54% of their patients with knee OA to specific exercise [14]. The conservative treatment of OA has been found to be insufficiently applied [9–11, 14]. A survey among patients attending an orthopaedic consultation in a public hospital in Australia showed that one-third of the patients had not received previous conservative, non-pharmacological management [12].

Projects, such as the ‘Swiss Learning Health System’ (www.slhs.ch), aim to solve this problem and attempt to improve Knee OA Management in Switzerland by e.g., supporting the use of international clinical guidelines for OA [46].

These findings indicate that measures are necessary to increase the level of programme awareness among potential patients, referring medical doctors and other stakeholders. To improve musculoskeletal care for OA patients, the current management of musculoskeletal care needs to be reframed and a comprehensive approach, ranging from education strategies to public health measures, is needed [9, 44, 47].

### Study strengths and limitations

The strength of this study is its focus on the clinical experiences of PTs and the combination of a theory-based and explorative approach in the online survey. This was achieved through combining both, the MIDI and open-ended questions.

The response rate of 61% can be considered to be good [48] and lies within the normal range of response rates from e-mail-based (25–70%) [48] and web-based surveys (20–47%) [49]. However, the number of answers per item varied because filter functions were used in the survey. Further, all valid answers, including those from incomplete surveys, were reported to not loose relevant information.

The responding PTs showed a notable heterogeneity regarding the degree of experience with the GLA:D® Switzerland programme. We have evaluated and integrated also the responses of PTs who had not performed a programme for patients, as their perspectives provide additional information. They were able to contribute their perspectives on acceptance and the facilitators and barriers, as these could have an impact on implementation.

The PTs who had not started programmes for patients still showed a high completion rate of the survey, which might be explained by the integrated filter functions or, simply, by high motivation to complete.

The age and gender distributions of the sample represent the general population of PTs in Switzerland quite well [50]. PTs holding an academic degree were somewhat over-represented. An explanation for this might be that the sample represents a group of early adopters and innovators.

The ability to generalise the findings is limited, however, since it can be assumed that the survey participants are systematically different from non-respondents [51]. The collected data reflects what was reported by the PTs. Those with positive attitudes and experiences of the GLA:D® Switzerland programme may be overrepresented in this sample. However, barriers were identified, and critical voices were more thoroughly captured using the open-ended questions.

## Conclusion

The acceptance, the practicality, and the identified facilitators are encouraging for the further successful roll-out of the GLA:D® Switzerland programme. The development of strategies that focus on the identified barriers and measures to increase the programme awareness can help to ensure a successful implementation of the GLA:D® Switzerland programme. This, in turn, will enhance the goal of increasing conservative, non-pharmacological treatment of knee and hip osteoarthritis in Switzerland based on current clinical guidelines.

### Electronic supplementary material

Below is the link to the electronic supplementary material.

Supplementary Material 1

## Data Availability

All data generated or analysed during this study are included in this published article and its supplementary information files.

## Abbreviations

GLA: D®:Good Life with Osteoarthritis in Denmark
IQR: Interquartile range
MIDI: Measurement Instrument for Determinants of Innovations
OA: Osteoarthritis
PT(s): Physiotherapist(s)
SD: Standard deviation
