# Supplementary material for: The feasibility, facilitators, and barriers in the initial implementation phase of ‘good life with osteoarthritis in Denmark’ (GLA:D®) in Switzerland: a cross-sectional survey
Source: BMC Health Serv Res. 2023 Sep 27;23:1034. doi: 10.1186/s12913-023-10023-7 (PMC10537542; doi:10.1186/s12913-023-10023-7)
Supplement: Supplementary file 1 — Supplementary Material 1 [file 12913_2023_10023_MOESM1_ESM.docx]

ADDITIONAL FILES

**Additional file 1**

Online-Survey

**Welcome! Bienvenue! Benvenuti!**

For the survey in German

Pour l'enquête en français

Al indadine in italiano

Thank you for taking the time to participate in the survey!

By doing so, you are making a valuable contribution to the implementation of GLA:D^®^ Switzerland.

The questions relate to the feasibility of GLA:D^®^ Switzerland and potential facilitators and barriers to its implementation in practice.

Your answers and your practical experience are very important and will help us to successfully implement the GLA:D^®^ Switzerland programme in Switzerland.

Your information will be collected anonymously by ZHAW, and no conclusions about your identity can be drawn.

Your participation in the survey is voluntary.

Answering the questions will take approximately 15 minutes.

Thank you for your support!

Instructions:

- You can navigate between survey pages using the "Back" and "Next" navigation buttons. Please do not use your browser's navigation (e.g., Internet Explorer).
- If you need to pause the survey and continue later, you must use the same laptop or PC and the same web browser. Otherwise, your data will not be saved, and the survey will start from the beginning.

What is your role related to GLA:D^®^ Switzerland?

I am...

- … GLA:D^®^ Switzerland trainer exclusively involved in the scientific field.
- … GLA:D^®^ Switzerland trainer involved in the clinical field.
- … a certified GLA:D^®^ Switzerland physiotherapist.
- *Filter: "GLA:D^®^ Switzerland trainer exclusively involved in the scientific field."*

*Thank you for your willingness to participate in the survey. However, for the survey, we only want to include GLA:D^®^ Switzerland trainers or GLA:D^®^ Switzerland certified physiotherapists who are working in a clinical setting. Thank you for your contribution!*

The following questions pertain to the implementation of the GLA:D^®^ Switzerland programme and its feasibility.

Have you already started a GLA:D^®^ Switzerland programme for patients?

- Yes
- No

How did you manage the marketing for your GLA:D^®^ Switzerland programme?

- Very well
- Well
- Neither nor
- Not well
- Not well at all
- Haven't done any marketing yet
- *Filter: These questions will not be displayed if "Haven't done any marketing yet" is selected.*

How did you advertise your GLA:D^®^ Switzerland programme?

Multiple selections possible.

- Internal recruitment
- Newsletter for physicians
- Information events
- Newspaper advertisement
- Flyer
- Short brochure
- Business cards
- Posters/roll-ups
- Social media
- via the clinical practice website
- Advertisement video
- Other:

Do you have any comments or suggestions for improving the marketing of the GLA:D^®^ Switzerland programme?

- *Filter: Have you already started a GLA:D^®^ Switzerland programme for patients? Answer ‘No’*

I have not started a GLA:D^®^ Switzerland programme for patients because...

Please state your reasons.

- *Filter: Have you already started a GLA:D^®^ Switzerland programme for patients? Answer ‘Yes’*

How many GLA:D^®^ Switzerland programmes for patients have you already conducted?

Please specify the total number of completed and currently ongoing programmes, as well as the total number of completed and current participants.

- Total number of programmes:
- Total number of participants:

How were patients included in your GLA:D^®^ Switzerland programme? (Ongoing and completed programmes)

- Number referred by a doctor (prescription):
- Number self-referred (self-payers):
- *All participants see this section.*

Do you intend to offer GLA:D^®^ Switzerland programmes for patients in the next six months?

- Yes. How many:
- No

How satisfied are you as a physiotherapist with the overall concept of the GLA:D^®^ Switzerland programme?

- Very satisfied
- Satisfied
- Neither nor
- Dissatisfied
- Very dissatisfied
- Comment:
- *Filter: Do you intend to offer GLA:D^®^ Switzerland programmes for patients in the next six months? Answer ‘No’*

I do not intend to conduct a GLA:D^®^ Switzerland programme in the next six months because...

Please state your reasons.

- *Filter: Have you already started a GLA:D^®^ Switzerland programme for patients? Answer ‘Yes’*

How did you, as a physiotherapist, manage the following activities?

|  | Not performed yet | Very poor | Poor | Partly poor, partly good | Good | Very good |
| --- | --- | --- | --- | --- | --- | --- |
| Conduct the first individual session |  |  |  |  |  |  |
| Conduct the second individual session |  |  |  |  |  |  |
| Conduct the third individual session |  |  |  |  |  |  |
| Register yourself in the GLA:D^®^ Switzerland register |  |  |  |  |  |  |
| Generate the login for the patient in the GLA:D^®^ Switzerland register |  |  |  |  |  |  |
| Register the patient and create a new case in the ‘RehabNET Test-Battery’ |  |  |  |  |  |  |
| Data entry in the ‘RehabNET Test-Battery’ (Filling out the electronic forms 1-3; medical history, diagnostic findings, clinical tests, and discharge) |  |  |  |  |  |  |

| Do you have any comments or suggestions for the activities mentioned above, or explanations for your answers? |
| --- |
|  |

How did you, as a physiotherapist, manage the following activities?

|  | Not performed yet | Very poor | Poor | Partly poor, partly good | Good | Very good |
| --- | --- | --- | --- | --- | --- | --- |
| Conducting the clinical tests: (second and fourth individual session) |  |  | | | | |
| - 30sec Chair Stand Test |  |  |  |  |  |  |
| - 40m Fast-paced Walk Test |  |  |  |  |  |  |
| - One leg hop Test |  |  |  |  |  |  |
| Conducting the consultation and instruction (patient education) |  |  |  |  |  |  |
| Conducting the neuromuscular exercise programme |  |  |  |  |  |  |
| Selecting and monitoring exercises with the exercise diary |  |  |  |  |  |  |
| Obtaining the patient’s declaration of consent for possible research projects using the collected data |  |  |  |  |  |  |
| Creating a report (Report Test-Battery) for the referring physician |  |  |  |  |  |  |

| Do you have any comments or suggestions for the activities mentioned above, or explanations for your answers? |
| --- |
|  |

Electronic data collection: In your opinion, how well were patients able to independently complete their digital questionnaires at entry (T1) and at discharge (T2)?

Multiple selections possible.

- Very good
- Good
- Partly poor/partly good
- Not good
- Not good at all
- Not performed yet
- I mostly completed the questionnaire on the PC/laptop/tablet together with the patients.
- I have often returned to a paper questionnaire.
- Comments:
- *Filter: Have you already started a GLA:D® Switzerland programme for patients? Answer ‘No’; This question is hidden.*

What worked well in the implementation of the GLA:D^®^ Switzerland programme?

- *All see this section.*

What challenges did you face in implementing the GLA:D^®^ Switzerland programme?

What would you recommend to others to avoid these challenges?

The following questions pertain to possible barriers and facilitators for the implementation of the GLA:D^®^ Switzerland programme. The questions are based on the "Measurement Instrument for Determinants of Innovations" by Fleuren et al. (2014).

Select the answer that best applies to your situation.

Definition: My institution = this refers to your workplace, such as a physiotherapy practice, a clinic, or a rehabilitation centre.

|  | Totally Disagree | Disagree | Neither nor | Agree | Totally agree |
| --- | --- | --- | --- | --- | --- |
| GLA:D^®^ Switzerland clearly describes the activities I should perform and in what order. |  |  |  |  |  |
| GLA:D^®^ Switzerland provides all necessary information and documents. |  |  |  |  |  |

**What additional information or documents would you have liked?**

|  | Totally Disagree | Disagree | Neither nor | Agree | Totally agree |
| --- | --- | --- | --- | --- | --- |
| There are components of the GLA:D^®^ Switzerland programme that are too complex. |  |  |  |  |  |

**Which components of the GLA:D^®^ Switzerland programme do you find too complex?**

|  | Totally Disagree | Disagree | Neither nor | Agree | Totally agree |
| --- | --- | --- | --- | --- | --- |
| GLA:D^®^ Switzerland can be easily integrated into my usual way of working. |  |  |  |  |  |
| The GLA:D^®^ Switzerland programme is important for hip and knee osteoarthritis patients. |  |  |  |  |  |

How much do you agree or disagree with the following statements regarding personal advantages or disadvantages of the GLA:D^®^ Switzerland programme?

|  | Totally disagree | Disagree | Neither nor | Agree | Totally agree |
| --- | --- | --- | --- | --- | --- |
| It is easier for me to treat patients according to current national and international clinical guidelines for osteoarthritis. |  |  |  |  |  |
| The GLA:D^®^ Switzerland programme facilitates my collaboration with other professionals (such as physicians). |  |  |  |  |  |
| I have more variety in my daily work. |  |  |  |  |  |
| There are financial benefits for me. |  |  |  |  |  |
| I have significantly more administrative work. |  |  |  |  |  |
| I have to work too much according to external requirements. |  |  |  |  |  |
| The application of the GLA:D^®^ Switzerland programme is too time-consuming for me. |  |  |  |  |  |
| The programme restricts me too much in the way I treat my patients. |  |  |  |  |  |
| Other: |  |  |  |  |  |

# How likely do you consider the following effects of the GLA:D^®^ Switzerland programme for your patients?

|  | Definitely not | Probably not | Maybe | Probably | Definitely |
| --- | --- | --- | --- | --- | --- |
| Improved quality of life |  |  |  |  |  |
| Less limitations in daily activities |  |  |  |  |  |
| Less pain |  |  |  |  |  |
| Less intake of pain medications |  |  |  |  |  |
| Fewer days of sick leave |  |  |  |  |  |
| More physical activity in everyday life |  |  |  |  |  |
| Patients can better manage their knee or hip problems on their own |  |  |  |  |  |
| Other: |  |  |  |  |  |

|  | Totally disagree | Disagree | Neither nor | Agree | Totally agree |
| --- | --- | --- | --- | --- | --- |
| I believe, as a physiotherapist, that it is important to record the examination findings, as well as questionnaires and clinical tests in the GLA:D^®^ Switzerland Register. |  |  |  |  |  |
| My patients are satisfied with the GLA:D^®^ Switzerland programme. |  |  |  |  |  |

|  | Totally disagree | Disagree | Neither nor | Agree | Totally agree |
| --- | --- | --- | --- | --- | --- |
| I am confident that I can implement the GLA:D^®^ Switzerland programme according to the specifications. |  |  |  |  |  |
| I know enough to implement the GLA:D^®^ Switzerland programme. |  |  |  |  |  |
| During the GLA:D^®^ Switzerland certification course, I got sufficient information about the content of GLA:D^®^ Switzerland. |  |  |  |  |  |

# To what extent does your work environment expect you to implement the GLA:D^®^ Switzerland programme?

- Very strongly
- Very
- Neither nor
- Not much
- Not at all

Has the management of the institution where you work made formal arrangements or measures for the implementation of the GLA:D^®^ Switzerland programme? (Room planning, adjustment of work schedules, certification of staff, etc.)?

- Yes
- No

|  | Totally disagree | Disagree | Neither nor | Agree | Totally agree |
| --- | --- | --- | --- | --- | --- |
| In my institution there is sufficient staff who can support the GLA:D^®^ Switzerland programme. |  |  |  |  |  |
| There are sufficient financial resources available to implement the GLA:D^®^ Switzerland programme. |  |  |  |  |  |
| I have enough time available in my institution to implement the GLA:D^®^ Switzerland programme |  |  |  |  |  |
| There are enough materials and facilities available in my institution to implement the GLA:D^®^ Switzerland programme. |  |  |  |  |  |

Is there someone in your institution responsible for coordinating the process of implementing GLA:D^®^ Switzerland? (Yourself or someone else?).

- Yes
- No

Are there organizational changes in your institution that could affect the implementation of GLA:D^®^ Switzerland? (Reorganization, merger, budget cuts, personnel changes, other innovations)

- Yes
- No

|  | Totally disagree | Disagree | Neither nor | Agree | Totally agree |
| --- | --- | --- | --- | --- | --- |
| I have easy access to information about the realisation of GLA:D^®^ Switzerland. |  |  |  |  |  |
| GLA:D^®^ Switzerland is well compatible with existing laws and regulations, such as the accounting with the health insurance. |  |  |  |  |  |

In the following, we will ask questions about your background and professional environment. Please select the answer that best applies to your situation.

| Year of birth | ….. | |
| --- | --- | --- |
| Sex: | - male | - female |
| In what type of institution do you work? (Multiple choices possible) | - Outpatient practice - Clinic / Hospital - Rehabilitation clinic - Interprofessional health centre - Institution for chronically ill - Other institution, please specify: … | |
| How many physiotherapists work in the institution where you are employed? | Please specify the number: | |
| Which of the following professional activities and job titles most closely apply to you? (Multiple choices possible) | - Employed physiotherapist - Practice trainer / Medical practice trainer - Specialist Manager / Professional content expert - Management function (Team leader, practice manager, department head) - Practice owner - Management board / clinic management - Other roles, please specify:… | |
| What is your highest completed physiotherapy education? | - No Bachelor's degree or subsequent title acquisition - Subsequent title acquisition - Bachelor of Science - Master of Science - Doctorate - Other, please specify:… | |
| For how many years have you been working as a physical therapist? | Please specify the number of years: | |
| Which GLA:D^®^ Switzerland certification course did you attend? | - April 2019, German-speaking Switzerland - Mai 2019, German-speaking Switzerland - September 2019, German-speaking Switzerland - September 2019, Ticino - Oktober 2019, Romandy | |

Is there anything else you would like to share with us? Please provide any additional feedback, suggestions, comments, or criticisms regarding the GLA:D^®^ Switzerland programme, its implementation, or this online survey.

Thank you very much for your participation and valuable contribution!

**Additional file 2**

**TABLE I**: Completion rate & time.

| **Completion rate (n=95)** | **N (%)** |
| --- | --- |
| Didn't start the survey | 9 (9.5%) |
| Didn't finish the survey | 21 (22.1%) |
| Finished | 56 (58.9%) |
| Finished after a break | 9 (9.5%) |
| **Completion time (minutes) (n=56)** |  |
| mean, ±SD (min-max) | 19.0 ±11.1 (5-61) |
| median | 16.5 |

**TABLE II:** Characteristics of responding PTs. Responses to the open text field ‘other’.

| **Characteristic** | **N (%)** |
| --- | --- |
| **Highest degree of education (n=65)** |  |
| **Other:** | **4 (6.2%)** |
| *CAS (n=1)* |  |
| *MAS Msk (n=2)* |  |
| *MScPT (n=1)* |  |
| **Place of work (n=65) ^a^** |  |
| **Other:** | **4 (5.0%)** |
| *‘Fitnesscenter’ (n=1)* |  |
| *‘Group practice of orthopaedists and physiotherapists’ (n=1)* |  |
| *‘With MTT / course / fitness centre included’ (n=1)* |  |
| *(additionally) ‘University’ (n=1)* |  |
| **Professional position / Professional activities / Job title (n=65) ^a^** |  |
| **Other:** | **3 (4.1%)** |
| *‘Self-employed physiotherapist’ (n=3)* |  |
| ^a^ Multiple answer possible | |

**TABLE III:** Intent to continue use.

| **‘Do you intend to offer GLA:D^®^ Switzerland programmes for patients in the next six months?’** | |
| --- | --- |
| **How many?** | |
|  | **N (%)** |
| **1-2** | 30 (68.2%) |
| **3-4** | 12 (27.3%) |
| **5-10** | 2 (4.5%) |
| Further answers to the open text field:  ‘Depending on demand / number of registrations’ (n=7) | |
| ‘3 groups per week’ (n=1) | |
| ‘Every week one group’ (n=1) | |
| ‘?’ (n=2) | |
| ‘I don’t know’ (n=1) | |
| **I do not intend to conduct a GLA:D^®^ Switzerland programme in the next six months because ... (n=5)** | |
| 1) ‘questions of organisation and feasibility’,  2) too much effort  3) too much unpaid effort for patient recruitment and too expensive flyer material  4) reluctance work with the PC on such a large extent  5) dissenting personal preferences regarding the selected exercises. | |

**TABLE IV:** Means used for advertising

| **Means used for advertising (n=59) ^a^** | **N (%)** |
| --- | --- |
| Internal recruitment | 49 (23.8%) |
| Newsletter for doctors | 37 (18.0%) |
| Informative meeting | 12 (5.8%) |
| Newspaper advertisement | 9 (4.4%) |
| Flyer | 34 (16.5%) |
| Short brochure | 12 (5.8%) |
| Social media | 9 (4.4%) |
| Via the practice website | 35 (17.0%) |
| Advertising film / commercial | 2 (1.0%) |
| Other: | 7 (3.4%) |
| *‘Professional training for doctors in our region’* | |
| *‘Direct contact with a doctor’* | |
| *‘Direct communication with doctors’* | |
| *‘Info to other physio practices in the region that do not (yet) perform GLA:D^®^’* | |
| *‘Newsletter for patients, Letters for doctors’* | |
| *‘Personal introduction of the programme to doctors’* | |
| *‘Gymnastics club’* | |
| ^a^ Multiple answer possible | |

**Additional file 3**

**TABLE V:** Coding matrix, open-ended questions

| **Category: Acceptability** | | |
| --- | --- | --- |
| "To what extent is a new idea, programme, process or measure judged as suitable, satisfying, or attractive to programme deliverers? To programme recipients?" [29]  To what extent do physiotherapists judge the GLA:D^®^ Switzerland programme, or elements of it, to be suitable, satisfactory or attractive. | | |
| **Subcategories** | **Positive (N)** | **Negative (N)** |
| **Deductive** | | |
| Satisfaction (with the programme in general) | **8** | . |
| Attractivity (of the programme in general) | . | 1 |
| A measure or activity is perceived as suitable / not suitable | . | 2 |
| **Inductive** | | |
| Required effort (When asked about satisfaction, too much effort is stated as limiting for satisfaction, otherwise effort is assigned to practicality (time required, scope) or barrier: innovation (effort, complexity)) | . | **8** |
| Satisfaction with / Attractivity of material (flyer, exercise diary, report) | 3 | 3 |
| Satisfaction: Exercise programme (content) | 1 | 4 |
| Satisfaction; Patient education (in general) | 1 | . |
| Satisfaction: Certification course | . | 1 |
| Satisfaction / Attractivity: Patient education repetitive content | . | 3 |
| Suitability: Content of the programme (e.g. lack of passive treatments, mobilisation exercises) | . | 2 |

| **Category: Practicality** | | | | |  |
| --- | --- | --- | --- | --- | --- |
| "To what extent can an idea, programme, process, or measure be carried out with intended participants using existing means, resources, and circumstances and without outside intervention?" [29] | | | | |  |
| **Subcategories** | **Positive (N)** | | **Negative (N)** | |  |
| **Deductive** | | | | |  |
| **Marketing** | 3 | | **8** | |  |
| **Patient recruitment** | 2 | | **9** | |  |
| **Digital patient questionnaire** | . | | **16** | |  |
| **Individual sessions** | 4 | | **6** | |  |
| **Data register** | 1 | | **23** | |  |
| Clinical tests & assessments | 4 | | . | |  |
| **Patient education** | 2 | | **6** | |  |
| **Exercise programme** | **15** | | **13** | |  |
| Obtain the patients declaration of consent | . | | 1 | |  |
| **Inductive** | | | | |  |
| **The GLA:D^®^ Switzerland programme in general** (process and implementation) | **11** | | 1 | |  |
| Required effort (An activity is described as too time-consuming or too extensive; also responses to item 'd4_complexity', are assigned to practicality, if they are described in terms of time or extent). | . | | 4 | |  |
| **Planning & Organisation** | 1 | | **24** | |  |
| Order process for material via the website | 1 | | . | |  |
| Group sessions | 1 | | . | |  |
| **Category: Facilitators & Barriers** | | | | | |
| - Facilitator: A factor described as facilitating on the use / implementation of GLA:D^®^ Switzerland. - Barrier: A factor described as impending on the use / implementation of GLA:D^®^ Switzerland.[17,25] | | | | | |
| **Subcategories** | | **Facilitator**  **(N)** | | **Barrier**  **(N)** | |
| **The Innovation** (The GLA:D^®^ Switzerland programme) | | | | | |
| **Deductive** | | | | | |
| Correctness | | . | | 4 | |
| Completeness | | 3 | | 4 | |
| Compatibility | | . | | 1 | |
| Observability | | 2 | | 1 | |
| **Complexity** | | . | | **30** | |
| **Inductive** | | | | | |
| ‘Complexity’ named issues: | |  | |  | |
| Planning / Organisation (extent, complexity) | | . | | 1 | |
| Realisation / Implementation (in general) | | . | | 1 | |
| Administration | | . | | 1 | |
| Accounting | | . | | 1 | |
| **Data register** | | . | | **22** | |
| Exercice programme (content: stretching exercices) | | . | | 1 | |
| Digital patient questionnaire | | . | | 2 | |
| Marketing / Advertising | | . | | 1 | |
| **The user** (GLA:D^®^ Switzerland certified PTs) | | | | | |
| **Deductive** | | | | | |
| Social Support | | 3 | | . | |
| Knowledge | | 1 | | . | |
| **Inductive** | | | | | |
| Personal likes or dislikes (e.g. ‘dislike of working with a computer’) | | . | | 4 | |
| **The patient** | | | | | |
| **Deductive** | | | | | |
| Patient satisfaction | | 1 | | 1 | |
| **Patient cooperation** | | 4 | | **11** | |
| **Patient age** | | . | | **6** | |
| **Inductive** | | | | | |
| **Patient motivation** | | **11** | | . | |
| **Patient skills (e.g. digital competencies)** | | . | | **6** | |
| Homogeneity / Heterogeneity of groups | | 1 | | 1 | |
| Other patient characteristics (e.g. profile of patient in general) | | . | | 1 | |
| **The organisational context** | | | | | |
| **Deductive** | | | | | |
| Staff capacity | | . | | 1 | |
| Financial resources | | . | | 1 | |
| **Time resources** | | . | | **8** | |
| **Material resources and facilities** | | . | | **13** | |
| Unsettled organisation | | . | | 2 | |
| **Inductive** | | | | | |
| Lack of or limited resources (not further specified) | | . | | 2 | |
| **The socio-political context** | | | | | |
| **Inductive** | | | | | |
| **Awareness level of the programme** | | . | | **11** | |
| **Patient recruitment** | | . | | **6** | |
| **Patient referral** (lack of patient referral or difficulty in recruiting patients through medical doctors) | | . | | **4** | |
| **No requests from patients / insufficient number of patients** (to run the programme cost covering) | | . | | **9** | |
| Cooperation of medical doctors | | 2 | | 3 | |
| Cooperation of other stakeholders | | . | | 2 | |
| Accounting via the health insurance company | | . | | 2 | |
| Scepticism about the use and confidentiality of electronic patient characteristics | | . | | 1 | |
| **Profitability** | | . | | **16** | |

**TABLE VI:** Reasons for non-implementation. Open-ended questions.

| **I have not yet started a GLA:D^®^ Switzerland programme for patients because…** | |
| --- | --- |
| **The Innovation (The GLA:D^®^ Switzerland programme)** | |
| Completeness: missing material in Italian | 2 |
| Compatibility: no group classes/programs are conducted in their own practice. | 1 |
| **The user (GLA:D^®^ Switzerland certified PTs, patients)** | |
| Personal preferences or aversions: Reluctance to work with a computer | 1 |
| **The organisational context** | |
| Lack of resources | **9** |
| lack of resources | 2 |
| facility | 2 |
| time | 4 |
| staff capacity | 1 |
| Unsettled organisation | 2 |
| **The socio-political context** | |
| Insufficient number of patients | **12** |
| Lack of patient referral | 2 |
| No requests from patients/insufficient number of patients | 5 |
| Insufficient number of patients to cover programme costs. | 4 |
| Patient recruitment (Difficulty recruiting patients through physicians.) | 1 |

**Additional file 4**

**TABLE VII:** Facilitators and barriers. Responses to the items based on the MIDI [26].

| **Innovation (GLA:D^®^ Switzerland)** | |  | **Valid n** | **Median** | **Disagree / totally disagree**  (%) | **Neutral**  (%) | **Agree / totally agree**  (%) |
| --- | --- | --- | --- | --- | --- | --- | --- |
| ***1. Procedural clarity*** | GLA:D^®^ Switzerland clearly describes the activities I should perform and in which order. |  | 69 | 5 | 2.9 | 1.4 | ***95.7*** |
| ***3. Completeness*** | GLA:D^®^ Switzerland provides all the information and documents needed to work with it properly. |  | 68 | 4 | 4.4 | 4.4 | ***91.2*** |
| **4. Complexity*** | There are components of the GLA:D^®^ Switzerland programme that are too complex. |  | 67 | 2 | 53.7 | 13.4 | **32.8** |
| 5. Compatibility | GLA:D^®^ Switzerland combines well with my usual way of working. |  | 66 | 4 | 12.1 | 12.1 | 75.8 |
| **7. *Relevance for***  ***patient*** | The GLA:D^®^ Switzerland programme is important for patients with hip and knee osteoarthrosis. |  | 68 | 5 | 7.4 | 4.4 | ***88.2*** |
| **Adopting user (GLA:D^®^ Switzerland certified PTs)** | |  |  |  |  |  |  |
| 8. Personal benefits& drawbacks | |  |  |  |  |  |  |
| Personal benefits: |  |  |  |  |  |  |  |
| guidelines | It is easier for me to treat patients according to current national and international clinical guidelines for osteoarthritis. |  | 65 | 4 | 7.7 | 21.5 | 70.8 |
| **cooperation** | The GLA:D^®^ Switzerland programme facilitates collaboration with other professionals (such as doctors). |  | 66 | 3 | **22.7** | 42.4 | 34.8 |
| variety | I have more variety in my daily work. |  | 65 | 4 | 10.8 | 15.4 | 73.8 |
| **financial benefits** | The use of the GLA:D^®^ Switzerland programme brings financial benefits for me. |  | 66 | 2 | **60.6** | 28.8 | 10.6 |
| Personal drawbacks: |  |  |  |  |  |  |  |
| **administrative effort*** | I have much more administrative work. |  | 66 | 4 | 9.1 | 21.2 | **69.7** |
| **foreign specifications*** | I have to work too much to external specifications. |  | 66 | 3 | 47.0 | 28.8 | **24.2** |
| **time-consuming*** | The use of the GLA:D^®^ Switzerland programme is too time-consuming for me. |  | 66 | 3 | 42.4 | 31.8 | **25.8** |
| **restrictions *** | The GLA:D^®^ Switzerland programme restricts me too much in the way I treat my patients. |  | 67 | 2 | 55.2 | 20.9 | **23.9** |
|  |  |  |  |  | **Probably not / Definitely not** | **Neutral** | **Probably /**  **Definitely** |
|  |  |  |  |  | (%) | (%) | (%) |
| ***9.*** ***Outcome***  ***expectations*** | Improved quality of life |  | 65 | 4 | 1.5 | 9.2 | ***89.2*** |
|  | Fewer restrictions in activities of daily living |  | 65 | 4 | 0.0 | 9.2 | ***90.8*** |
|  | Less pain |  | 65 | 4 | 3.1 | 36.9 | 60.0 |
|  | Less intake of painkillers |  | 65 | 4 | 0.0 | 24.6 | 75.4 |
|  | Fewer days of sick leave |  | 64 | 4 | 7.8 | 28.1 | 64.1 |
|  | Increased levels of physical activity in everyday life |  | 65 | 5 | 1.5 | 12.3 | ***86.2*** |
|  | Patients can better manage their knee or hip problems on their own |  | 65 | 4 | 1.5 | 9.2 | ***89.2*** |
| ***10. Professional***  ***obligation*** | I think, as a physiotherapist, that it is important to record the examination results as well as questionnaires and clinical tests in the GLA:D^®^ Switzerland Register. |  | 63 | 5 | 3.2 | 9.5 | ***87.3*** |
| 11. Patient satisfaction | My patients are satisfied with the GLA: D^®^ Switzerland programme. |  | 60 | 4 | 1.7 | 26.7 | 71.7 |
| ***12. Patient***  ***cooperation*** | Patients who are participating in the GLA: D^®^ Switzerland programme are motivated and cooperative. |  | 64 | 4 | 1.6 | 17.2 | ***81.3*** |
| 13.a. Social support | I can count on adequate support from the GLA: D^®^ Switzerland organisation when it comes to realising the GLA:D^®^ Switzerland programme. |  | 63 | 4 | 12.7 | 20.6 | 66.7 |
| 13.b. Social support | I can count on adequate support from the management of my institution when it comes to realising the GLA:D^®^ Switzerland programme. |  | 63 | 4 | 11.1 | 20.6 | 68.3 |
|  |  |  |  |  | **Not much /**  **Not at all** | **Neutral** | **Very /**  **Very strongly** |
|  |  |  |  |  | (%) | (%) | (%) |
| **15. Subjective norm** | To what extent does your work environment expect you to implement the GLA:D^®^ Switzerland programme? |  | 65 | 3 | **21.5** | 29.2 | 49.2 |
| ***16. Self-efficacy*** | I am confident that I can implement the GLA:D^®^ Switzerland programme according to the specifications. |  | 64 | 5 | 4.7 | 1.6 | ***93.8*** |
| ***17. Knowledge*** | I know enough to implement the GLA:D^®^ Switzerland programme. |  | 65 | 5 | 0.0 | 3.1 | ***96.9*** |
| ***18. Awareness of content of innovation*** | During the GLA:D^®^ Switzerland certification course, I got sufficient information about the content of GLA:D^®^ Switzerland. |  | 65 | 5 | 3.1 | 4.6 | ***92.3*** |
| **Organisational context** | |  |  |  |  |  |  |
| **19. Formal ratification**  **by management**** | Has the management set up formal arrangements in your institution relating to the use of GLA:D^®^ Switzerland (room planning, work plans, etc.)? |  | 65 | . | **26.2** | . | 73.8 |
| **21. Staff capacity** | In my institution there is sufficient staff who can support the GLA:D^®^ Switzerland programme. |  | 65 | 4 | **32.3** | 3.1 | 64.6 |
| 22. Financial resources | There are sufficient financial resources available to implement the GLA:D^®^ Switzerland programme. |  | 65 | 4 | 16.9 | 27.7 | 55.4 |
| **23. Time available** | In my institution I have enough time available to implement the GLA:D^®^ Switzerland programme. |  | 65 | 4 | **23.1** | 23.1 | 53.8 |
| ***24. Material resources***  ***and facilities*** | There are enough materials and space in my institution to implement the GLA:D^®^ Switzerland programme. |  | 65 | 4 | 9.2 | 10.8 | ***80.0*** |
| ***25. Coordinator***** | In my institution, I, or someone else, is responsible for coordinating the process of implementing GLA:D^®^ Switzerland. |  | 65 | . | 18.5 | . | ***81.5*** |
| **26. Unsettled**  **organisation***** | Are there any organisational changes in your institution that could affect the implementation of GLA:D^®^ Switzerland? (Reorganisation, merger, reduction, change of staff or other innovations) |  | 65 | . | **23.1** | . | 76.9 |
| ***27. Accessible***  ***Information*** | I have easy access to information about the realisation of GLA:D^®^ Switzerland. |  | 64 | 4 | 9.4 | 9.4 | ***81.3*** |
| **Socio-political context** | |  |  |  |  |  |  |
| 29. Legislation and  regulations | GLA:D^®^ Switzerland is well compatible with existing laws and regulations, such as the accounting with the health insurance company. |  | 63 | 4 | 7.9 | 31.7 | 60.3 |
| Numbers and items in **bold** represent a **barrier** (≥ 20% disagree / totally disagree) and in ***bold*** and ***italic*** a ***facilitator*** (≥ 80% agree / totally agree) reported by the GLA:D^®^ Switzerland certified PTs. The numeration of the items is based on the original numeration of the MIDI [26]. *: reversed scaling; **: dichotomous answer categories 1 ‘no’ and 2 ‘yes’.; ***: dichotomous answer categories 1 ‘yes’ and 2 ‘no’. | | | | | | | |
